# De novo and inherited *TCF20* pathogenic variants are associated with intellectual disability, dysmorphic features, hypotonia, and neurological impairments with similarities to Smith–Magenis syndrome

**DOI:** 10.1186/s13073-019-0623-0

**Published:** 2019-02-28

**Authors:** Francesco Vetrini, Shane McKee, Jill A. Rosenfeld, Mohnish Suri, Andrea M. Lewis, Kimberly Margaret Nugent, Elizabeth Roeder, Rebecca O. Littlejohn, Sue Holder, Wenmiao Zhu, Joseph T. Alaimo, Brett Graham, Jill M. Harris, James B. Gibson, Matthew Pastore, Kim L. McBride, Makanko Komara, Lihadh Al-Gazali, Aisha Al Shamsi, Elizabeth A. Fanning, Klaas J. Wierenga, Daryl A. Scott, Ziva Ben-Neriah, Vardiella Meiner, Hanoch Cassuto, Orly Elpeleg, J. Lloyd Holder, Lindsay C. Burrage, Laurie H. Seaver, Lionel Van Maldergem, Sonal Mahida, Janet S. Soul, Margaret Marlatt, Ludmila Matyakhina, Julie Vogt, June-Anne Gold, Soo-Mi Park, Vinod Varghese, Anne K. Lampe, Ajith Kumar, Melissa Lees, Muriel Holder-Espinasse, Vivienne McConnell, Birgitta Bernhard, Ed Blair, Victoria Harrison, The DDD study, Donna M. Muzny, Richard A. Gibbs, Sarah H. Elsea, Jennifer E. Posey, Weimin Bi, Seema Lalani, Fan Xia, Yaping Yang, Christine M. Eng, James R. Lupski, Pengfei Liu

**Affiliations:** 1 Baylor Genetics, Houston, TX 77021 USA; 2 Northern Ireland Regional Genetics Service, Belfast City Hospital, Belfast, UK; 3 Department of Molecular and Human Genetics, Baylor College of Medicine, Houston, TX 77030 USA; 4 Nottingham Genetics Service, Nottingham City Hospital, Nottingham, UK; 5 Department of Pediatrics, Baylor College of Medicine, San Antonio, TX 78207 USA; 6 North West Thames Regional Genetics Service, 759 Northwick Park Hospital, London, UK; 7 Human Genome Sequencing Center, Baylor College of Medicine, Houston, TX 77030 USA; 8 Dell Children’s Medical Group, Austin, TX 78723 USA; 9 Division of Genetic and Genomic Medicine, Nationwide Children’s Hospital; and Department of Pediatrics, College of Medicine, Ohio State University, Columbus, OH 43205 USA; 10 Department of Pediatrics, College of Medicine & Health Sciences, United Arab University, Al Ain, UAE; 11 Department of Pediatrics, Tawam Hospital, Al-Ain, UAE; 12 Department of Pediatrics, Section of Genetics, University of Oklahoma Health Sciences Center, Oklahoma City, OK 73104 USA; 13 Department of Human Genetics and Metabolic Diseases, Hadassah-Hebrew University Medical Center, Jerusalem, Israel; 14 Department of Pediatrics, Texas Children’s Hospital, Houston, TX 77030 USA; 15 Department of Pediatrics, University of Hawaii, Honolulu, HI 96826 USA; 16 Centre de Génétique Humaine, Université de Franche-Comté, Besançon, France; 17 Department of Neurology, Boston Children’s Hospital, Boston, MA 0211 USA; 18 Gene DX, Gaithersburg, MD 20877 USA; 19 West Midlands Regional Clinical Genetics Service and Birmingham Health Partners; and Women’s and Children’s Hospitals NHS Foundation Trust, Birmingham, UK; 20 East Anglia Regional Genetics Service, Addenbrooke’s Hospital, Cambridge, UK; 21 All-Wales Medical Genetics Service, University Hospital of Wales, Cardiff, UK; 22 South East of Scotland Clinical Genetic Service, Western General Hospital, Edinburgh, UK; 23 North East Thames Regional Genetics Service, Great Ormond Street Hospital, London, UK; 24 South East Thames Regional Genetics Service, Guy’s Hospital, London, UK; 25 Oxford Regional Genetics Service, Oxford University Hospitals, Oxford, UK; 26 Wessex Clinical Genetics Service, Princess Anne Hospital, Southampton, UK; 27 The DDD Study, Wellcome Trust Sanger Institute, Hinxton, Cambridge, UK; 28 The Hebrew University of Jerusalem, Jerusalem, Israel; 29 Monique and Jacques Roboh Department of Genetic Research, Hadassah-Hebrew University Medical Center, 91120 Jerusalem, Israel; 30 Present address: Department of Medical and Molecular Genetics, Indiana University School of Medicine, Indianapolis, IN 46202 USA; 31 Department of Molecular Physiology and Biophysics, Baylor College of Medicine, Houston, TX 77030 USA; 32 Present address: Mayo Clinic Florida, Department of Clinical Genomics, Jacksonville, FL 32224 USA

**Keywords:** *TCF20*, 22q13, Neurodevelopmental disorders, Smith–Magenis syndrome, Haploinsufficiency, Loss-of-function variants, Deletions

## Abstract

**Background:**

Neurodevelopmental disorders are genetically and phenotypically heterogeneous encompassing developmental delay (DD), intellectual disability (ID), autism spectrum disorders (ASDs), structural brain abnormalities, and neurological manifestations with variants in a large number of genes (hundreds) associated. To date, a few de novo mutations potentially disrupting *TCF20* function in patients with ID, ASD, and hypotonia have been reported. *TCF20* encodes a transcriptional co-regulator structurally related to *RAI1*, the dosage-sensitive gene responsible for Smith–Magenis syndrome (deletion/haploinsufficiency) and Potocki–Lupski syndrome (duplication/triplosensitivity).

**Methods:**

Genome-wide analyses by exome sequencing (ES) and chromosomal microarray analysis (CMA) identified individuals with heterozygous, likely damaging, loss-of-function alleles in *TCF20*. We implemented further molecular and clinical analyses to determine the inheritance of the pathogenic variant alleles and studied the spectrum of phenotypes.

**Results:**

We report 25 unique inactivating single nucleotide variants/indels (1 missense, 1 canonical splice-site variant, 18 frameshift, and 5 nonsense) and 4 deletions of *TCF20*. The pathogenic variants were detected in 32 patients and 4 affected parents from 31 unrelated families. Among cases with available parental samples, the variants were de novo in 20 instances and inherited from 4 symptomatic parents in 5, including in one set of monozygotic twins. Two pathogenic loss-of-function variants were recurrent in unrelated families. Patients presented with a phenotype characterized by developmental delay, intellectual disability, hypotonia, variable dysmorphic features, movement disorders, and sleep disturbances.

**Conclusions:**

*TCF20* pathogenic variants are associated with a novel syndrome manifesting clinical characteristics similar to those observed in Smith–Magenis syndrome. Together with previously described cases, the clinical entity of *TCF20*-associated neurodevelopmental disorders (TAND) emerges from a genotype-driven perspective.

**Electronic supplementary material:**

The online version of this article (10.1186/s13073-019-0623-0) contains supplementary material, which is available to authorized users.

## Background

The human chromosome 22q13 region is involved with various genetic and genomic disorders, including Phelan–McDermid syndrome (MIM 606232), in which terminal deletion of 22q13.3 encompassing the critical gene *SHANK3* is frequently observed [1]. Occasionally, deletions proximal to the classical Phelan–McDermid syndrome region have been reported, affecting chromosome 22q13.2 without directly disrupting *SHANK3* [2–4]. It remains unknown whether the abnormal neurodevelopmental phenotypes observed in patients with 22q13.2 deletions are caused by dysregulation of *SHANK3* or haploinsufficiency of previously undefined “diseases genes” within the deletion. Recently, a bioinformatics analysis of genes within 22q13.2 highlighted that *TCF20* and *SULT4A1* are the only two genes within this region that are predicted to be highly intolerant to loss-of-function (LoF) variants and are involved in human neurodevelopmental processes [5]. In particular, *TCF20* was predicted to be of higher intolerance to LoF variants as reflected by its higher pLI (probability of LoF intolerance) score (pLI = 1), making it the most promising candidate disease gene underlying neurodevelopmental traits associated with 22q13.2 deletion disorders.

**Fig. 1:**
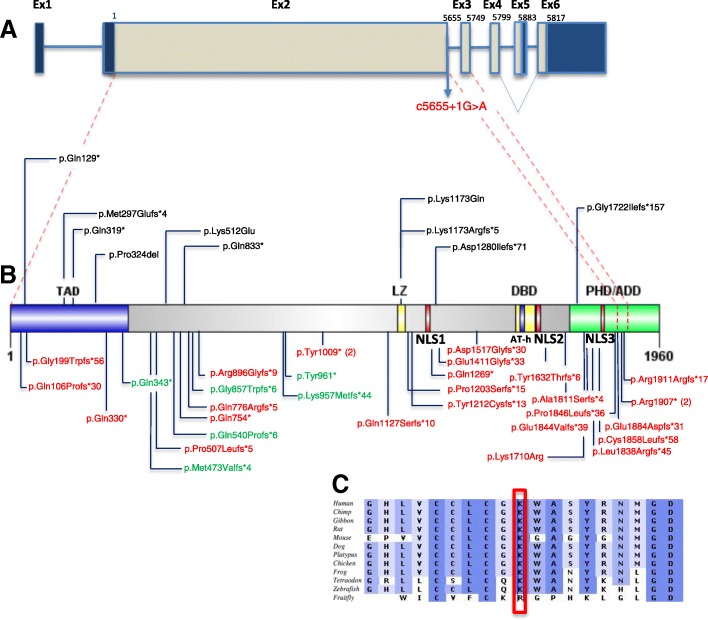
*TCF20* gene, protein domain structure, and location of mutations. **a** Schematic representation of *TCF20*, exons are shown to scale with the coding sequence in gray and untranslated regions in dark blue. There is an in frame stop codon in the alternatively spliced exon 5 generating a shorter isoform (referred as isoform 2) (Genbank: NM_181492.2) lacking exon 5 in the 3′ coding region. The position of the first coding nucleotide is shown in exon 2, numbers above boxes indicate cDNA numbering at last nucleotides of exon boundaries or last nucleotide of stop codons. Red dashed lines show the exon boundaries relative to the amino acid position shown in **b**. **b** Domain structures of TCF20 with the mutations currently identified. Protein domains are indicated above or below the structure. Abbreviations as follows: TAD, transactivation domain; NLS, nuclear localization signals; LZ, leucine zipper; DBD, DNA-binding domain; AT-h, AT-hook domain; PHD/ADD, Plant Homeodomain/ADD. In red and below the protein structure are the mutations identified in this study. In black and above the protein structure are the mutations previously reported (see text). All the de novo SNVs detected in anonymized subjects presenting with mild to severe neurodevelopmental disorder from our cohort are represented in green and located below the protein structure. All the mutations occur before the last exon of *TCF20.* In parentheses are indicated the number of times the recurring variants are observed. **c** ClustalW multi-species alignment obtained with Alamut software of the region containing Lys1710Arg showing the high level of conservation of the mutated residue. Intensities of shades of blue are proportional to the degree of cross-species conservation

*TCF20* (encoding a protein previously known as SPRE-binding protein, SPBP) is composed of six exons, which encode two open reading frames of 5880 or 5814 nucleotides generated by alternative splicing. The shorter isoform (referred to as isoform 2, Genbank: NM_181492.2) lacks exon 5 in the 3′ coding region. Isoform 1 (Genbank: NM_005650.3) is exclusively expressed in the brain, heart, and testis and predominates in the liver and kidney. Isoform 2 is mostly expressed in the lung ([6, 7]; Fig. 1). TCF20 was originally found to be involved in transcriptional activation of the *MMP3* (matrix metalloproteinase 3, MIM 185250) promoter through a specific DNA sequence [8]. More recently, it has been shown to act as a transcriptional regulator augmenting or repressing the expression of a multitude of transcription factors including *SP1* (specificity protein 1 MIM 189906), *PAX6* (paired box protein 6, MIM 607108), *ETS1* (E twenty-six 1, MIM 164720), *SNURF* (*SNRPN* upstream reading frame)/*RNF4* (MIM 602850), and *AR* (androgen receptor, MIM 313700) among others [9–11]. *TCF20* is widely expressed and shows increased expression in the developing mouse brain particularly in the hippocampus and cerebellum [12, 13]. Babbs et al. studied a cohort of patients with autism spectrum disorders (ASDs) and proposed *TCF20* as a candidate gene for ASD based on four patients with de novo heterozygous potentially deleterious changes, including two siblings with a translocation disrupting the coding region of *TCF20*, one frameshift and one missense change in another two patients [6]. Subsequently, Schafgen et al. reported two individuals with de novo truncating variants in *TCF20* who presented with intellectual disability (ID) and overgrowth [14]. In addition, pathogenic variants in *TCF20* have also been observed in two large cohort studies with cognitive phenotypes of ID and developmental delay (DD) [15, 16]. These isolated studies clearly support a role for *TCF20* as a disease gene. However, a systematic study of patients with *TCF20* pathogenic variant alleles from a cohort with diverse clinical phenotypes is warranted in order to establish a syndromic view of the phenotypic and molecular mutational spectrum associated with a *TCF20* allelic series.

Interestingly, *TCF20* shares substantial homology with a well-established Mendelian disease gene, *RAI1*, which is located in human chromosome 17p11.2 (MIM 607642). LoF mutations or deletions of *RAI1* are the cause of Smith–Magenis syndrome (SMS; MIM 182290), a complex disorder characterized by ID, sleep disturbance, multiple congenital anomalies, obesity, and neurobehavioral problems [17–21], whereas duplications of *RAI1* are associated with a developmental disorder characterized by hypotonia, failure to thrive, ID, ASD, and congenital anomalies [22, 23], designated Potocki–Lupski syndrome (PTLS; MIM 610883). Recent studies suggested that *TCF20* and *RAI1* might derive from an ancestral gene duplication event during the early history of vertebrates [9]. Therefore, it is reasonable to hypothesize that, as paralogous genes, mutations in *TCF20* may cause human disease by biological perturbations and molecular mechanisms analogous to those operative in *RAI1-*mediated SMS/PTLS.

In this study, we describe the identification of *TCF20* pathogenic variations by either clinical exome sequencing (ES) or clinical chromosomal microarray analysis (CMA) from clinically ascertained subjects consisting of cohorts of patients presenting with neurodevelopmental disorders as the major phenotype as well as with various other suspected genetic disorders. We report the clinical and molecular characterization of 28 subjects with *TCF20* de novo or inherited pathogenic single nucleotide variants/indels (SNV/indels) and 4 subjects with interstitial deletions involving *TCF20.* These subjects present with a core phenotype of DD/ID, dysmorphic facial features, congenital hypotonia, and variable neurological disturbances including ataxia, seizures, and movement disorders; some patients presented features including sleep issues resembling those observed in SMS. Additionally, we report the molecular findings of 10 anonymized subjects with pathogenic *TCF20* SNVs or deletion/duplication copy-number variants (CNVs). We demonstrate that ascertainment of patients from clinical cohorts driven by molecular diagnostic findings (*TCF20* LoF variants) delineates the phenotypic spectrum of a potentially novel syndromic disorder*.*

## Methods

### Subjects

The study cohort consists of 31 unrelated families including one family with a set of affected monozygotic twins; four affected heterozygous parents from these families are also included. All the affected individuals were recruited under research protocols approved by the institutional review boards of their respective institutions after informed consent was obtained. Subject #17 who received clinical exome sequencing evaluation at Baylor Genetics presented with hypotonia, autism spectrum disorder, and behavioral abnormalities. Six additional patients carrying SNV/indels (subjects #1, #6, #11, #13, #17, #20, and #25) were identified retrospectively from the Baylor Genetics exome cohort of > 11,000 individuals after filtering for rare potential LoF variants in previously unsolved cases with overlapping neurological phenotypes. Subject #7 was recruited from Children’s Hospital of San Antonio (TX), and the pathogenic variant in *TCF20* was detected via diagnostic exome sequencing at Ambry Genetics (Aliso Viejo, CA, USA). Subjects #3 and #4 were recruited from the Hadassah Medical Center from Israel. Subjects #2, #5, #8, #9, #10, #12, #14, #15, #16, #18, #19, #21, #22, #23, #24, #26, #27, and #28 were identified through the DDD (Deciphering Developmental Disorders) Study in the UK.

Two patients (subjects #29 and #30) carrying deletion CNVs in chromosome 22q13 were identified in the Baylor Genetics CMA cohort of > 65,000 subjects. Subject #31 carrying a deletion of *TCF20* was recruited from the Decipher study. Subject #32 carrying a deletion encompassing 11 genes including *TCF20* was recruited from Boston Children’s Hospital through microarray testing from GeneDX. These cases with positive CNV findings did not receive exome sequencing evaluation.

**Fig. 2:**
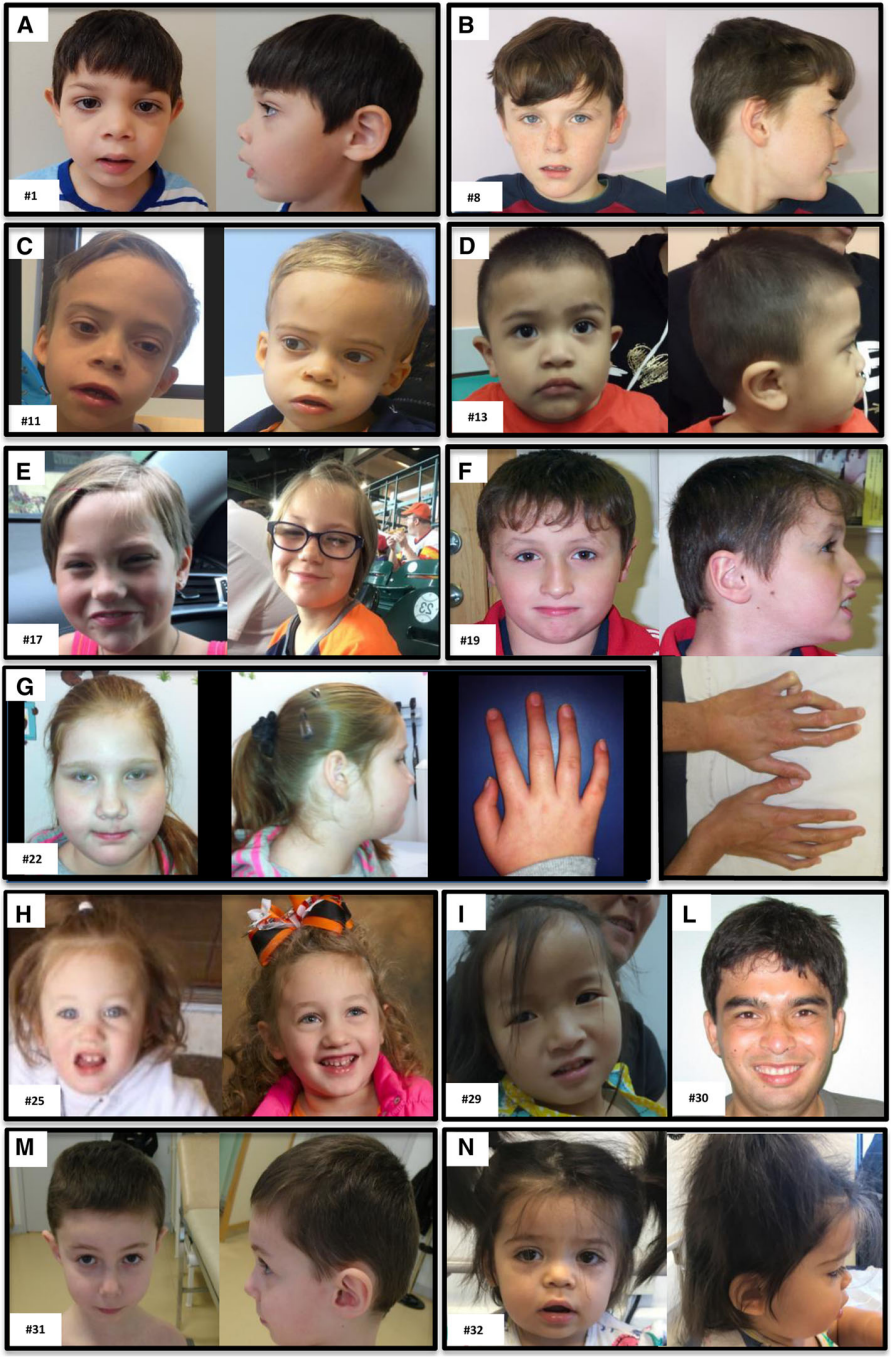
Twelve individuals with TCF20-associated neurodevelopmental disorder (TAND). Facial features are variable from normal or mildly dysmorphic: subject #8 (**b**), subject #25 (**h**), subject #29 (**i**), and subject #31 (**m**) to dysmorphic: macrocephaly in subjects #11 (**c**) and #30 (picture taken at 22 years old) (**l**); brachycephaly in subject #19 (**f**); midface hypoplasia in subject #17 and #32 (**e**, **n**); long eyelashes, thick lips, and occipital grove in subject #32 (**n**); upper lip abnormality including tented or thin upper lip in subjects #1, #11, #13, and #17 (**a**, **c**, **d**, **e**); coarse facies in subjects #1 and #11 (**a**, **c**); long face, full cheeks, deep-set eyes, and prominent lower lip in subject #22 (**g**). Digital anomalies include contracture of the fifth finger in subject #19 (**f**) and slender fingers in subject #22 (**g**)

All participating families provided informed consent via the procedures approved under the respective studies to which they were recruited. The parents or legal guardians of subjects shown in Fig. 2 provided consent for publication of photographs.

### Molecular analysis

Clinical ES analysis was completed for subjects #1, #6, #11, #13, #17, #20, and #25 in the exome laboratory at Baylor Genetics and was conducted as previously described [24]. Samples were also analyzed by cSNP array (Illumina HumanExome-12 or CoreExome-24 array) for quality control assessment of exome data, as well as for detecting large copy-number variants (CNVs) and regions of absence of heterozygosity [25, 26].

The ES-targeted regions cover > 23,000 genes for capture design (VCRome by NimbleGen®), including the coding and the untranslated region exons. The mean coverage of target bases was 130X, and > 95% of target bases were covered at > 20X [24]. PCR amplification and Sanger sequencing to verify all candidate variants were done in the proband and the parents when available, according to standard procedures, and candidate variants were annotated using the *TCF20* RefSeq transcript NM_005650.3. Exome sequencing and data analysis for the DDD study were performed at the Wellcome Sanger Institute as previously described [16]. Sequencing and data analysis at the Hadassah Medical Center and Ambry Genetics were conducted as previously described [27, 28].

The two CNV deletions were detected using customized exon-targeted oligo arrays (OLIGO V8, V9, and V10) designed at Baylor Genetics [29–31], which cover more than 4200 known or candidate disease genes with exon-level resolution. The deletion in subject #32 was detected by a customized Agilent 180k array, which provides interrogation of 220 regions of microdeletion/microduplication syndrome and 35 kb backbone. The deletion in subject #31 from the Decipher study was detected by the Agilent 180k array.

### RNA studies to evaluate for potential escape from nonsense-mediated decay (NMD) associated with the *TCF20* alleles with premature stop codons

Total cellular RNA was extracted from peripheral blood according to the manufacturer’s protocol. After DNase I treatment to remove genomic DNA (Ambion), cDNA was synthesized from oligo dT with SuperScript III Reverse Transcriptase (Invitrogen). Primers were designed to span multiple exons of *TCF20* to amplify the target variant site from cDNA. The amplified fragments were sized and Sanger sequenced to ensure that cDNA rather than genomic DNA was amplified. Negative controls were also set up without reverse transcriptase to confirm that there was no genomic DNA interference. Sanger sequencing results were analyzed for the ratio of mutant allele versus wild type allele to infer whether there was an escape from nonsense-medicated decay.

## Results

### Phenotypic spectrum

Table 1 summarizes the clinical findings in the 32 subjects; further details can be found in Additional file 1: Clinical information. Twenty individuals are male, 12 are female, and at the last examination, ages ranged from 1 to 20 years. Additionally, an affected biological parent of subjects #1, #5, and #7 and twins #27 and #28 were found to be carriers of the *TCF20* pathogenic variants and their ages ranged from 42 to 47 years (these are not listed in the tables but briefly described in text Additional file 1: Clinical information). Five individuals (#2, #8, #10, #19, and #26) from the DDD cohort previously reported in a large study with relatively uncharacterized neurodevelopmental disorder [16] have been included in this study after obtaining more detailed clinical information.

Overall, the majority of the subjects included in our cohort presented with a shared core phenotype of motor delay (94%, *n* = 30/32), language delay (88%, *n* = 28/32), moderate-to-severe ID (75%, *n* = 24/32), and hypotonia (66%, *n* = 21/32). Some of the variable features reported in the patients include ASD/neurobehavioral abnormalities (66%, *n* = 21/32), movement disorder (44%, *n* = 14/32), sleep disturbance (38%, *n* = 12/32), seizures (25%, *n* = 8/32), structural brain abnormalities (22%, *n* = 7/32), growth delay and feeding problems (13%, *n* = 4/32), macrocephaly (25%, *n* = 8/32), digital anomalies (34%, *n* = 11/32), otolaryngological anomalies (3/32, 9%), and inverted nipples (13%, *n* = 4/32) (Tables 1 and 2 and Additional file 1: Clinical information). Facial dysmorphisms (78%, *n* = 25/32) were also variable and included anomalies reminiscent of SMS such as a tented or protruding upper lip in a subset of the patients (16%, *n* = 5/32) and the affected mother of subject #5, brachycephaly (9%, *n* = 3/32), and midface hypoplasia (6%, *n* = 2/32) (Tables 1 and 2, Additional file 1: Clinical information, and Fig. 2).

**Table 1:** Phenotypic data in individuals with *TCF20* mutations

Subject	Age/sex	ID	Neurobehavioral abnormalities	Dysmorphic facial features	Sleep disturbance	Macrocephaly	Overgrowth	Digital anomalies	Seizures	Motor delay	Hypotonia	Movement disorder	Language delay	Structural brain abnormalities	Other features, additional variants detected
#1	3 y M	+	NR	Coarse facies, wide nasal bridge, long eyelashes, tented upper lip	NR	−	−	NR	Nocturnal epilepsy	+	NR	NR	+	NR	Duplicated left kidney, atrial septal defect, tethered spinal cord, dilated coronary artery
#2^a^ DDD_269612	14.3 y F	+	NR	Frontal bossing, full cheeks	NR	−	+	Long fingers with contracture of 5th finger, long toes	NR	+	NR	NR	+	NR	Joint hypermobility, increased carrying angle
#3	20 y M	NR	ASD, ADHD, anxiety	NR	NR	Borderline	+	NR	NR	+	Congenital	Dyspraxia	+	NR	NR
#4	3.25 y M	NR	ASD	Frontal bossing, triangular face	+	+	NR	NR	NR	+	NR	Visuo spatial perception difficulty, dyspraxia	NR	NR	NR
# 5 DDD_277986	8.5 y M	Moderate	NR	Mild dysmorphic facial features with depressed nasal bridge	NR	−	−	NR	NR	Severe	+	NR	Severe	NR	Feeding difficulties (tube fed)
#6	10.2 y M	+	ASD	NR	NR	NR	−	NR	Complex partial intractable	+	Congenital	Ataxia	Severe expressive/mild receptive	Mild cerebellar atrophy	Growth retardation, short stature, type 2 fiber muscular atrophy, laryngeal cleft, recurrent otitis media
#7^b^	10 y M	+	ASD, ADHD, anxiety, self-harming behavior	Mild	−	−	−	Tapered 5th fingers with minor cutaneous 2–3 to syndactyly	Epileptic disorder with multifocal origin	+	Congenital generalized	NR	+	NR	De novo c.1189C>T, p.Gln397* in *SLC6A1*
#8^a^ DDD_274421	6.9 y M	+	ADHD	Abnormal hair whorl	+	−	NR	NR	+	+	NR	NR	+	NR	NR
#9 DDD_267241	11.9 y M	+	ADHD, obsessive–compulsive trait	Brachycephaly, low-set ears	NR	−	−	Finger-tip pads, sandal gap, clinodactyly of the 5th toe	NR	−	NR	Abnormal movement	−	NR	Inverted nipples, feeding difficulties, microcephaly
#10^a^ DDD_261665	11.5 y F	Moderate	Obsessive compulsive, food-seeking, aggression	Abnormal facial shape	+	+	Overweight	NR	NR	Moderate	+	NR	Severe receptive and expressive	NR	NR
#11	3.9 y M	+	ASD	Deep-set eyes, hypertelorism, long philtrum, tented upper lips, wide mouth, full lips, mild coarsening	+	+	−	NR	NR	+	Congenital generalized	Distal spasticity	Severe expressive/receptive	−	NR
#12 DDD_305239	3 y F	NR	ASD	USPFs, epicanthus, short nose, depressed nasal bridge, short lingual frenulum	+	−	−	NR	NR	+	NR	NR	**+**	NR	NR
#13	1.2 y M	+	NR	Plagiocephaly, epicantal folds, depressed nasal roots, tented upper lip	+	NR	NR	5th finger clinodactyly	NR	+	NR	NR	+	NR	NR
# 14 DDD_284745	7.9 y F	Mild	Hyperactivity	USPFs, anteverted nares	NR	−	−	5th finger clinodactyly	Febrile seizures	Mild	Mild	Motor coordination disorder	Mild	NR	Oligohydramnios on 20 weeks scan, ptosis, drooling, fatigue
# 15 DDD_274474	2.5 y F	NR	NR	Brachycephaly, myopathic facies, depressed nasal bridge, anteverted nares, open mouth with downturned angles	NR	−	−	NR	NR	+	+	Gait ataxia	**+**	NR	NR
#16 DDD_285718	6.5 y M	Mild	ASD, ADHD	NR	NR	−	−	NR	NR	Moderate	+	−	Moderate	+	NR
#17^b^	5.4 y F	+	ASD, self-injurious, aggression, hyperactivity, food-seeking behavior	Midface hypoplasia, bulbous nose, tented upper lips	+	−	−	NR	NR	+	Congenital generalized	NR	Severe expressive/receptive	NR	De novo c.1307G>T (p.R436L) in *ZBTB18*
#18 DDD_286061	9 y F	+	Hyperactivity	High anterior hairline, large and tall forehead, temporal hypotrichosis, low-set, posteriorly rotated ears, broad philtrum, narrow mouth	NR	−	−	Broad hallux, 2–3 toe syndactyly	NR	+	+	NR	**+**	NR	Strabismus, joint hypermobility, drooling
#19^a^ DDD_266071	10.2 y M	NR	ASD, hyperactivity	Brachycephaly	NR	−	Tall stature	Contracture of 5th finger	+	+	NR	Paroxysmal dyskinesia	+	NR	High-pitched voice, ichthyosis (due STS deficiency)
#20	8.1 y M	+	NR	−	+	NR	NR	NR	NR	Mild	Congenital generalized	Impaired coordination, increased tone at the elbows	Expressive	NR	NR
#21 DDD_304759	33.3 y M	Moderate	NR	NR	NR	−	Tall stature	NR	NR	NR	NR	NR	NR	NR	High-pitched voice
#22 DDD_299802	9.9 y F	Severe	NR	Deep-set eyes, full cheeks, long face, tall forehead, prominent lower lip and chin, sunken eyes	NR	+	+	Slender fingers	NR	+	+	Jerky movements	+	NR	Hypermetropia, amblyopia
#23 DDD_304749	4 y M	NR	ASD	Broad forehead, short nose, depressed nasal bridge	NR	−	−	NR	NR	+	NR	NR	**+**	+	Inverted nipples
#24 DDD_300882	7.8 y M	NR	ASD	Low-set ears	NR	−	−	NR	+	+	NR	NR	+	NR	NR
#25	5.3 y F	+	ASD	Mild	+	−	−	NR	NR	Mild	Congenital generalized	NR	Severe expressive	NR	Hypohidrosis
#26 ^a^ DDD_261626	3.1 y F	NR	ASD	Long eyelashes, DSPFs, epicanthic folds, everted lower lip, open mouth	NR	−	−	Tapered fingers	NR	+	+	NR	NR	Delayed CNS myelination and lack of cerebral white matter	NR
#27 DDD_294521	17 y M	Moderate	−	Plagiocephaly, bilateral ptosis, horizontal crus of helix, malar flattening, narrow mouth	NR	−	Truncal obesity	Short, tapering fingers with incurved 5th fingers, mild bilateral hallux valgus	−	Mild	+	Motor coordination difficulties	+	Right posterior plagiocephaly	Inverted nipples
#28 DDD_294521.	17 y M	Moderate	−	Plagiocephaly, bilateral ptosis, horizontal crus of helix, malar flattening, narrow mouth	NR	−	Truncal obesity	Short, tapering fingers with incurved 5th fingers, mild bilateral hallux valgus	−	Mild	+	Motor coordination difficulties	+	Right posterior plagiocephaly	Inverted nipples, High arched palate
#29	4 y F	+	Repetitive behavior, ASD,ADHD	NR	+	−	−	NR	NR	Mild	Congenital generalized	NR	Mixed severe expressive/receptive	NR	NR
#30	14 y M	+	active autistic disorder	+	+	+	NR	NR	NR	Mild	Congenital generalized	NR	+	NR	Scoliosis
#31	5 y M	+	NR	NR	NR	+	+	NR	+	+	Congenital generalized	Balance disorder	+	NR	NR
#32	1.1 y F	+	Autistic features, food-seeking behavior	Midface hypoplasia, long eyelashes, thick lips, occipital groove	+	+	NR	NR	NR	+	Mild generalized	Mild spasticity in ankle dorsiflexors	Significant expressive/receptive	Cavum septum pellucidum and vergae	Dysphagia, GERD, renal cyst, delayed visual maturation
Schafgen et al. [14]; Babbs et al. [6]; Lelieveld et al. [15]; McRae et al. [16]*	*n* = 2 (12–14 y, M); *n* = 4 (*n* = 3F, 1 M); *n* = 6 (NR)	Mild to moderate (*n* = 8/12)	Autistic features (*n* = 5/12), stereotypic behavior (*n* = 1/12), aggression (*n* = 1/12)	*n* = 4/12	NR	*n* = 3/12	*n* = 2/12	*n* = 1/12	*n* = 1/12	*n* = 5/12	*n* = 3/12	NR	*n* = 5/12	Abnormality of the cerebrum *n* = 2/12	Inverted nipples (*n* = 2/12), tapered fingers (*n* = 1/12), small penis (*n* = 1/12), abnormal eye physiology (*n* = 1/12), abnormality of the mouth (*n* = 1/12)

*Abbreviations*: *ID* intellectual disability, *M* male, *F* female, *y* year old, *NR* not reported, *N/A* not applicable, + present feature, − absent feature, *ASD* autism spectrum disorder, *ADHD* attention-deficit hyperactivity disorder, *FTT* failure to thrive, *USPF* upslanting palpebral fissures, *DSPF* downslanting palpebral fissures, *IUGR*, intrauterine growth retardation, *GERD* gastro-esophageal reflux disorder

^a^Five patients from the original McRae et al. DDD cohort [16] (individual #2, #8, #10, #19, and #26) were included in this study

^b^The clinical phenotype in subjects #7 and #17 might result from a combination of *TCF20* variants and additional contributions in variants detected in *SLC6A1* and *ZBTB18*, respectively

*Two additional patients from this study were included in the meta-analysis from previous studies

**Table 2:** Comparison of clinical presentation in this study and in the published cohort

Clinical features	Number of subjects in this study*	Percentage in this study	Number of subjects in the published cohorts (Schafgen et al. [14]; Babbs et al. [6]; Lelieveld et al. [15]; McRae et al. [16]**)	Percentage in the published cohort
ID	24/32	75	8/12	67
Neurobehavioral abnormalities	21/32	66	7/12	58
Dysmorphic facial features	25/32	78	4/12	33
Sleep disturbance	12/32	38	NR	NR
Macrocephaly	8/32	25	3/12	25
Overgrowth/obesity/tall stature	9/32	28	2/12	17
Digital anomalies	11/32	34	1/12	8
Seizures	8/32	25	1/12	8
Motor delay	30/32	94	5/12	42
Hypotonia	21/32	66	3/12	25
Movement disorder	14/32	44	NR	NR
Language delay	28/32	86	5/12	42
Structural brain abnormalities	7/32	22	2/12	17

*Abbreviations*: *ID* intellectual disability, *NR* not reported

*Five patients from the original McRae et al. DDD cohort [16] (individual #2, #8, #10, #19, and #26) were included in this study

**Two additional patients from this study were included in the meta-analysis from previous studies

To date, deleterious variants in *TCF20* have been identified in cohorts of individuals with diverse neurodevelopmental disorders (NDDs) including ID (66%, *n* = 8/12), language delay (42%, *n* = 5/12), neurobehavioral abnormalities (58%, *n* = 7/12), hypotonia (25%, *n* = 3/12), one patient with seizures (*n* = 1/12, 8%), and macrocephaly/overgrowth (25%, *n* = 3/12) [14–16] (Tables 1, 2, and 3). In Babbs et al., the first study reporting *TCF20* as a potential disease gene, all four patients presented with ASD, three with ID and one of the patients with midface hypoplasia [6]. Of note, subject 1 of our cohort presented with mild delayed motor milestones, generalized hypotonia, and, in particular, dysmorphic features including midface hypoplasia, tented upper lips, along with sleep issues, ASD, food-seeking behavior, and aggressive behavior; these clinical features are similar to those reported in SMS [32–34]. In Schafgen et al., both patients presented with ID, developmental delay, relative macrocephaly, and postnatal overgrowth [14]. Postnatal overgrowth, overweight, and tall stature are seen in 4, 3, and 2 patients from our cohort, respectively. Patients that present with these three “growth acceleration” features account for 28% (9/32) of our cohort. Furthermore, we have observed sleep disturbance (38%, *n* = 12/32) and neurological features absent from previous published studies including ataxia/balance disorder (22%, *n* = 7/32), dyspraxia (6%, *n* = 2/32), dyskinesia/jerky movements (6%, *n* = 2/32), and peripheral spasticity (19%, *n* = 6/32) (Tables 1 and 2).

**Table 3:** *TCF20* (NM_005650.3) variants identified in the present study

Subject	Type of mutation	Coordinates hg19	Nucleotide change	Effect	Exon number	Inheritance	Additional variants
#1	Frameshift	g.42610999_42611002dupGTGG	c.310_313dupCCAC	p.Gln106Profs*30	2	Maternal	NR
#2	Frameshift	g.42610718dupA	c.594dupT	p.Gly199Trpfs*56	2	De novo	No
#3	Nonsense	g.42610324G>A	c.988C>T	p.Gln330*	2	Mother negative	No
#4	Frameshift	g.4260792delG	c.1520delC	p.Pro507Leufs*5	2	De novo	No
#5	Nonsense	g.42609052G>A	c.2260C>T	p.Gln754*	2	Maternal	No
#6	Frameshift	g.42608984_42608985delCT	c.2327_2328delAG	p.Gln776Argfs*5	2	De novo	NR
#7	Frameshift	g.42608627delC	c.2685delG	p.Arg896Glyfs*9	2	Maternal	de novo c.1189C>T, p.Gln397* in *SLC6A1*
#8	Nonsense	g.42608285A>T	c.3027T>A	p.Tyr1009*	2	De novo	No
#9	Nonsense	g.42608285A>T	c.3027T>A	p.Tyr1009*	2	Not known	No
#10	Frameshift	g.42607933delG	c.3379delC	p.Gln1127Serfs*10	2	De novo	No
#11	Frameshift	g.42607707dupC	c.3605dupG	p.Pro1203Serfs*15	2	Mother negative	NR
#12	Frameshift	g.42607678_42607679dupAC	c.3633_3634dupGT	p.Tyr1212Cysfs*13	2	De novo	No
#13	Nonsense	g.42607507G>A	c.3805C>T	p.Gln1269*	2	De novo	NR
#14	Frameshift	g.42607081dupC	c.4231dupG	p.Glu1411Glyfs*33	2	De novo	No
#15	Frameshift	g.42606763dupC	c.4549dupG	p.Asp1517Glyfs*30	2	De novo	No
#16	Frameshift	g.42606418delA	c.4894delT	p.Tyr1632Thrfs*6	2	De novo	No
#17	Missense	g.42606183 T>C	c.5129A>G	p.Lys1710Arg	2	De novo	de novo c.1307G>T (p.R436L) in *ZBTB18*
#18	Frameshift	g.42605882dupT	c.5430dupA	p.Ala1811Serfs*4	2	De novo	No
#19	Frameshift	g.42605800_42605801dupGC	c.5511_5512dupCG	p.Leu1838Argfs*45	2	De novo	No
#20	Frameshift	g.42605782_42605783dupCA	p.5529_5530dupTG	p.Glu1844Valfs*39	2	De novo	NR
#21	Frameshift	g.42605775delG	c.5537delC	p.Pro1846Leufs*36	2	Not known	No
#22	Frameshift	g.42605742dupC	c.5570dupG	p.Cys1858Leufs*58	2	De novo	No
#23	Frameshift	g.42605659_42605660delTC	c.5652_56553delGA	p.Glu1884Aspfs*31	2	Not known	No
#24	Canonical splicing	g.42605656C>T	c5655+1G>A	N/A	Intron 2	Not known	No
#25	Nonsense	g.42575645G>A	c.5719C>T	p.Arg1907*	3	De novo	NR
#26	Nonsense	g.42575645G>A	c.5719C>T	p.Arg1907*	3	De novo	No
#27	Frameshift	g.42575632delG	c.5732delC	p.Pro1911Argfs*17	3	Paternal	No
#28	Frameshift	g.42575632delG	c.5732delC	p.Pro1911Argfs*17	3	Paternal	No
#29	Del 22q13.2q13.3	g.42394098-45037128del	2.64 Mb DEL	Deletion of 37 genes	Whole gene	Not known (adopted)	No
#30	Del 22q13.2	g.42607466-42770878del	163 kb DEL	Deletion of Exon1	1	De novo	No
#31	Del 22q13.2	g.42488512-42616581del	128 kb DEL	Deletion of 3 genes	Whole gene	De novo	No
#32	Del 22q13.2	g.42373034-42776457del	403 kb DEL	Deletion of 11 genes	Whole gene	De novo	No
Shafgen et al. [14]	Nonsense (*n* = 1) Frameshift (*n* = 1)	N/A	N/A	N/A	2	De novo	No
Babbs et al. [6]	Complex chromosomal rearrangement (*n* = 2) Missense (*n* = 1) Frameshift (*n* = 1)	N/A	N/A	N/A	2/partial gene deletion	Possibly parental mosaicism/de novo	No
Lelieveld et al. [15]	Nonsense (*n* = 2) Frameshift (*n* = 2)	N/A	N/A	N/A	2/3	De novo	No
McRae et al. [16]*	Inframe deletion (*n* = 1) Missense variant (*n* = 1)	N/A	N/A	N/A	2	De novo	No

*Abbreviations*: *N/A* not applicable, *NR* not reported

*The original study reported 7 patients, 5 of which (#2, #8, #10, #19, and #26) have been included in this study with more detailed phenotypic characterization

### Genomic analyses

**Fig. 3:**
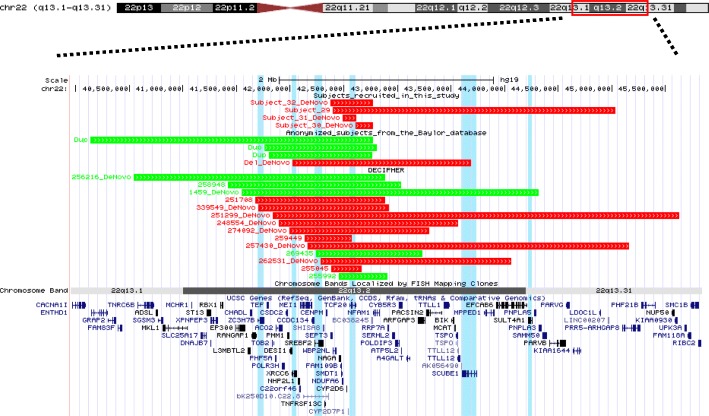
Schematic representation of 22q13.2 CNVs involving *TCF20* identified in this study and DECIPHER. Deletion intervals in the patients are indicated in red, whereas duplications are indicated in green. The four subjects that are clinically characterized in this study are shown on the top of the figure. Subjects #29, #31, and #32 have larger deletions encompassing multiple flanking genes not currently associated with disease. Subject 30 has a deletion encompassing solely *TCF20.* Anonymized subjects with CNVs affecting *TCF20* that are detected by exon-targeted CMA from the Baylor database are shown in the middle. Cases from DECIPHER with a CNV encompassing *TCF20* are shown in the bottom of the figure. Genes with a pLI score > 0.9 that are located within any of the deletions shown in this figure are highlighted by blue vertical segments. *ZC3H7B*, *XRCC6*, *SREBF2*, and *TCF20* have pLI scores > 0.99. *SCUBE1* and *SULT4A1* have pLI scores > 0.95

We detected a spectrum of variant types including 25 unique heterozygous SNVs/indels and 4 CNVs involving *TCF20* (Figs. 1 and 3)*.* The 25 variants include missense (*n* = 1), canonical splice-site change (*n* = 1), frameshift (*n* = 18), and nonsense changes (*n* = 5) (Table 3), and they are all located in exons 2 or 3 or the exon2/intron2 boundary of *TCF20*. All of these variants are absent in the Exome Aggregation Consortium and gnomAD (accessed September 2018) (Table 2, Fig. 1) databases. The variant c.5719C>T (p.Arg1907*) has been detected in both subjects #25 and #26 while c.3027T>A (p.Tyr1009*) is present in both subjects #8 and #9 (Table 2). Although recurring in unrelated subjects, neither of these two changes occurs within CpG dinucleotides. The missense mutation in codon 1710 (p.Lys1710Arg) in subject #17, which was confirmed by Sanger sequencing to have arisen de novo, is located in a highly conserved amino acid (Fig. 1c) within the PHD/ADD domain of TCF20 [9], and the substitution is predicted to be damaging by multiple in silico prediction tools including SIFT and Polyphen-2. In addition to this variant, another de novo c.1307G>T (p.Arg436Leu) missense variant in *ZBTB18* (MIM 608433; autosomal dominant mental retardation 22, phenotype MIM 612337) was found in this patient. A nonsense mutation in *ZBTB18* has been recently reported in a patient with ID, microcephaly, growth delay, seizures, and agenesis of the corpus callosum [35]. The c.1307G>T (p.Arg436Leu) variant in *ZBTB18* is also absent from ExAC and gnomAD databases and predicted to be damaging by Polyphen2 and SIFT and could possibly contribute to the phenotype in this patient, representing a potential blended (overlapping) phenotype due to a dual molecular diagnosis [36]. Interestingly, in addition to the c.2685delG (p.Arg896Glyfs*9) variant in *TCF20* inherited from the affected mother, subject #7 harbors also a de novo likely pathogenic variant (p.Gln397*) in *SLC6A1* that, as described for subject #17, could contribute to a blended phenotype in this patient. Defects in *SLC6A1* can cause epilepsy and developmental delay (MIM 616421), overlapping with the presentation observed and reported to date in patients with deleterious variants in *TCF20.* For all the other patients, the clinical test referenced in this study, either exome sequencing or microarray, did not detect additional pathogenic or likely pathogenic variants in other known disease genes underlying the observed neurodevelopmental disorder.

Sanger sequencing confirmed that subjects # 1 to #28 are heterozygous for the *TCF20* variants and showed that these changes were absent from the biological parents in 17 patients; in 4 families (subjects #1, #5, #7, and siblings #27 and #28), the variants were inherited from parents with a similar phenotype, confirming the segregation of the phenotype with the variant within the families (Table 2, Fig. 1, and Additional file 1: Clinical information). One or two of the parental samples were unavailable for study in six cases.

In addition to SNVs/indels, we have studied four patients with heterozygous interstitial deletions (128 kb to 2.64 Mb in size) that include *TCF20* (subjects #29 to #32, Fig. 3, Tables 1, 2, and 3). Subject #29 is a 4-year-old adopted female with global developmental delay, hypotonia, mixed receptive-expressive language disorder, ASD, ID, ADHD, and sleep disturbance. She was found to have a 2.64-Mb deletion at 22q13.2q13.31 involving *TCF20* and 36 other annotated genes. Subject #30 is a 14-year-old male with global psychomotor delay, ASD, severe language delay, macrocephaly, congenital hypotonia, scoliosis, and abnormal sleep pattern. A heterozygous de novo 163-kb deletion was found in this individual removing exon 1 of *TCF20*. Subject #31 is a 5-year-old male with developmental disorder, seizures, and balance disorder with a 128-kb de novo heterozygous deletion involving *TCF20*, *CYP2D6*, and *CYP2D7P1*. Subject #32 is a 13-month-old female with global developmental delay, hypotonia, and emerging autistic features with a 403-kb deletion encompassing 11 annotated genes including *TCF20.* The deletions in subjects #30, #31, and #32 do not contain genes other than *TCF20* that are predicted to be intolerant to loss-of-function variants, making *TCF20* the most likely haploinsufficient disease gene contributing to these patients’ phenotypes. In patient #29, two genes included in the deletion, *SCUBE1* and *SULT4A1*, have pLI scores of 0.96 and 0.97, respectively. These two genes may contribute to the phenotypic presentation of this patient together with *TCF20* (pLI = 1) (Fig. 3).

We have also observed additional individuals presenting with neurodevelopmental disorders of variable severity from our clinical database, carrying de novo truncating variants (*n* = 6, Fig. 1, in green), deletions (*n* = 1, de novo, Fig. 3), and duplications (*n* = 3, Fig. 3) involving *TCF20*. These individuals are included in this study as anonymized subjects (Figs. 1 and 3). Additionally, we observed nine deletions (six are de novo) and five duplications (five are de novo) spanning *TCF20* from the DECIPHER database; in some cases, the deletion CNV incorporates other potentially haploinsufficient genes (Fig. 3 and Additional file 1: Table S1). Taken together, these data from anonymized subjects combined with the current clinically characterized subjects in this study corroborate *TCF20* being associated with a specific Mendelian disease condition.

Our results indicate that all variants identified in subjects #1 to #32 and four affected carrier parents represent either pathogenic or likely pathogenic (the de novo missense variant in subject #17) alleles. We performed RNA studies in patients #11, #25, and #7 and in the affected mother and sister of patient #7, who all carry premature termination codon (PTC) *TCF20* variants that are expected to be subject to NMD as predicted by the NMDEscPredictor tool [37], because the PTCs are upstream of the 50-bp boundary from the penultimate exon based on both *TCF20* transcripts (NM_181492.2 and NM_005650.3). Our data suggest that the mutant *TCF20* mRNAs did not obey the “50-bp penultimate exon” rule and they all escaped from NMD (Additional file 1: Figure S2), which is consistent with a previous observation [6]. Despite this, we did not observe a clear genotype-to-phenotype correlation among the different mutation categories. For instance, patients with missense mutations or truncating mutations near the terminal end of the gene did not present with milder phenotypes when compared with patients carrying early-truncating mutations in *TCF20* or large deletion encompassing *TCF20* and surrounding several genes—the phenotype appears consistent.

## Discussion

We report 32 patients and 4 affected carrier parents with likely damaging pathogenic variants in *TCF20*. Phenotypic analysis of our patients, together with a literature review of previously reported patients, highlights shared core syndromic features of individuals with *TCF20*-associated neurodevelopmental disorder (TAND)*.* Previous reports have collectively associated deleterious variants in *TCF20* with ID, DD, ASD, macrocephaly, and overgrowth [6, 14–16] (Tables 1 and 2). The majority of the individuals in our cohort displayed an overlapping phenotype characterized by congenital hypotonia, motor delay, ID/ASD with moderate to severe language disorder, and variable dysmorphic facial features with additional neurological findings (Tables 1 and 2 and Fig. 2). We observe in our cohort that it is possible to have *TCF20* deleterious variants transmitting across generations in familial cases (subjects #1, #5, and #7 and the twin brothers #27 and #28; Table 1, Additional file 1: Clinical information). Our parent carriers presented with an apparently milder phenotype; the mother of subject #1 showed mild dysmorphic facial features; the mother of subject #5 had features including ID, prominent forehead, tented upper lip, and short nose.

It is intriguing that TCF20 contains regions of strong sequence and structural similarity to RAI1 (Additional file 1: Figure S1) [22, 38–41]. *RAI1* encodes a nuclear chromatin-binding multidomain protein with conserved domains found in many chromatin-associated proteins, including a polyglutamine and two polyserine tracts, a bipartite nuclear localization signal, and a zinc-finger-like plant homeodomain (PHD) (Additional file 1: Figure S1) [39]. A previous phylogenetic study of *TCF20* and *RAI1* suggested that a gene duplication event may have taken place early in vertebrate evolution, just after branching from insects, giving rise to *TCF20* from *RAI1*, this latter representing the ancestral gene [9]. The two proteins share organization of several domains such as N-terminal transactivation domain, nuclear localization signals (NLS), and PHD/ADD at their C-terminus (Additional file 1: Figure S1) [9]. The PHD/ADD domain associates with nucleosomes in a histone tail-dependent manner and has an important role in chromatin dynamics and transcriptional control [42]. Here, we report that some patients with *TCF20* mutations may present phenotypic features reminiscent of SMS such as craniofacial abnormalities which include brachycephaly, tented upper lips, midface hypoplasia, neurological disturbance (seizure, ataxia, abnormal gait), failure to thrive, food-seeking behaviors, and sleep disturbance.

To our knowledge, ataxia, hypertonia, food-seeking behavior, sleep disturbance, and facial gestalt reminiscent of SMS have not been previously reported in association with *TCF20* pathogenic variants and represent a further refinement of TAND. Interestingly, subject #17 who presented features reminiscent of SMS harbors a missense variant c.5129A>G (p.Lys1710Arg) in the F-box/GATA-1-like finger motif part of the PHD/ADD domain in TCF20. The PHD/ADD domain that maps between amino acid positions 1690–1930 of TCF20 is highly conserved in RAI1 and confers the ability to bind the nucleosome and function as a “histone-reader” (HR) [8, 9]. Interestingly, mutations occurring in the region of GATA-1-like finger of RAI1 (p.Asp1885Asn and p.Ser1808Asn), in close proximity to the corresponding region of TCF20 where p.Lys1710 lies, are also associated with SMS [38, 39, 43].

Postnatal overgrowth has been previously reported in two patients with *TCF20* defects [14]. We observe overgrowth, obesity, or tall stature in nine of the patients from our cohort. Interestingly, eight of these nine patients fall into an older age group (> 9.5 years old), representing 73% (8/11) of the patients older than 9.5 years old from our cohort; in the age group younger than 9.5 years old, only 6.7% (1/15) of them presented overgrowth. Further longitudinal clinical studies are warranted to dissect the etiologies of overgrowth, obesity, and tall stature, and to investigate whether these growth accelerations are age-dependent.

Of note, a subset of patients reported herein have sleep disturbance (38%, *n* = 12/32), hyperactivity (28%, *n* = 9/32), obsessive–compulsive traits (9%, *n* = 3/32), anxiety (6%, *n* = 2/32), and food-seeking behavior/early obesity (16%, *n* = 5/32) (Table 2), which could ultimately be attributed to circadian rhythm alterations as seen in SMS and PTLS [22, 38, 39]. Receptors for the steroid hormones estrogen (ER) and androgen (AR) have an emerging role in circadian rhythms and other metabolic function regulation in the suprachiasmatic nuclei in vertebrates through alteration of brain-derived neurotropic factor (*BNDF*) expression in animal models [44–47]. Interestingly, *Bdnf* is also downregulated in the hypothalamus of *Rai1*+/− mice, which are hyperphagic, have impaired satiety, develop obesity, and consume more food during light phase [48–50]. Since TCF20 has also been implicated in the regulation of ER- and AR-mediated transcriptional activity [10, 11, 51], we speculate that TCF20 might play a role in the regulation of circadian rhythms through steroid hormone modulation and disruption of its activity could lead to the phenotype observed in a subset of our patients.

Besides patient #17, all other patients carry either deletion or truncating variants occurring before the last exon of *TCF20* that are predicted to be loss-of-function either through presumably NMD or by truncating essential domains of the TCF20 protein (Fig. 1). The frameshifting mutations from patients #27 and #28 are expected to result in a premature termination codon beyond the boundary of NMD, therefore rendering the mutant protein immune to NMD [37]. Future studies are warranted to delineate the exact correlation between genotype and phenotype in light of the potential escape from NMD and the potential pathway overlapping and interaction between TCF20 and RAI1 in the determination of the phenotype. It has been shown that around 75% of mRNA transcripts that are predicted to undergo NMD escape destruction and that the nonsense codon-harboring mRNA may be expressed at similar levels to wild type [52]. Therefore, alternative to NMD, we can speculate that, for instance, the truncating mutations that occur earlier in the gene before the first NLS (amino acid position 1254–1268) (Fig. 1, Additional file 1: Figure S1) in subjects #1 to #12 may determine loss-of-function of TCF20 due to either decreased level of protein in the nucleus with consequent cytoplasmic accumulation and/or to the absence of key functional C-terminal domains including PHD/ADD domains and/or DBD, AT-hook, NLS2, and NLS3, these latter representing unique motifs not conserved between TCF20 and RAI1 (Fig. 1, Additional file 1: Figure S1). It has been previously shown that the frameshift mutation c.3518delA (p.Lys1173Argfs*5) in *TCF20* in one patient with ASD produces a stable mRNA that escapes NMD [6]. Data from our RNA studies corroborates this observation that *TCF20* alleles with premature termination codon mutations may in general escape NMD. However, it should also be noted that NMD and mRNA turn over may be tissue specific and the current tissue tested is limited to blood. Based on this hypothesis, the position of amino acid truncation, for example, within the NLS or DNA-binding domain, may contribute to the prediction of genotype–phenotype correlation. The truncated TCF20 protein may retain partial function, representing hypomorphic alleles, or act in a dominant-negative manner sequestering transcription factors and co-factors in the absence of transcriptional modulation. Another possibility is that, due to the similarity between RAI1 and TCF20, mutated products of *TCF20* could interfere with RAI1 pathways through the aforementioned mechanisms. Due to the complexity of the protein regulation and the variety of functional domains present in TCF20 (Additional file 1: Figure S1) that are not fully characterized, further studies are needed to refine the genotype–phenotype correlation.

Finally, although disorders associated with 22q13.2 deletions (encompassing *TCF20*) share similar features with Phelan–McDermid syndrome caused by deletion of *SHANK3*, our study provides evidence for the hypothesis that the major phenotypes observed in the former disorder are likely caused by direct consequence of *TCF20* defects. Phenotypes specific for *TCF20*, such as sleep disturbances and movement disorders, may help clinically distinguish the 22q13.2 deletions from the 22q13.3 deletions (*SHANK3*). It is tempting to hypothesize that dosage gain of *TCF20* may also be disease causing, given the similar observation at the 17p11.2 locus, where copy number gain of *RAI1* was found to cause PTLS, potentially presenting mirror trait endophenotypes in comparison to SMS (e.g., underweight versus overweight) [53, 54]. This hypothesis predicts that *TCF20* duplications are expected to cause similar neurodevelopmental defects as observed in the deletions, which is supported by the observation of *TCF20* duplications from anonymized individuals with neurodevelopmental disorders, some of which are de novo (Fig. 2 and Additional file 1: Figure S1); additionally, one may speculate that specific phenotypes caused by *TCF20* duplication may present mirror trait compared to those associated with the deletions, such as underweight versus overweight and schizophrenia spectrum disorders versus autism spectrum disorders. Further work is warranted to investigate the consequence of dosage gain of *TCF20* in human disease.

## Conclusions

Our findings confirm the causative role of *TCF20* in syndromic ID, broaden the spectrum of *TCF20* mutations recently reported, begin to establish an allelic series at this locus, and may help to understand the molecular basis of this new TAND syndrome. We also observe some patients with pathogenic variants in *TCF20* presenting phenotypes reminiscent of SMS, suggesting potential common downstream targets of both *TCF20* and *RAI1*. We suggest without molecular testing that it is challenging for a TAND diagnosis to be clinically reached purely based on the phenotypes observed in most patients. This underlines the importance of clinical reverse genetics for patients presenting with developmental delay and minor dysmorphic features, where positioning genotype-driven analysis (ES, CMA, or a combination of both) early in the “diagnostic odyssey” could improve the molecular diagnostic outcome and facilitate appropriate clinical management including recurrence risk counseling [55].

## Additional file

Additional file 1: Clinical information. Clinical presentation of the subjects in this study. **Table S1.** Phenotypes for de-identified subjects from the DECIPHER database. **Figure S1.** Schematic representation of key conserved domains between TCF20 and RAI1. **Figure S2.** *TCF20* alleles with premature termination codon variants escape from nonsense-mediated decay (NMD). (PDF 393 kb)

## Abbreviations

ASD: Autism spectrum disorder
CMA: Chromosomal microarray analysis
CNV: Copy-number variants
DD: Developmental delay
ES: Exome sequencing
ID: Intellectual disability
NDD: Neurodevelopmental disorders
NMD: Nonsense-mediated decay
PHD: Plant homeodomain
PTC: Premature termination codon
TAND: *TCF20*-associated neurodevelopmental disorders
