# Correction to: De novo and inherited *TCF20* pathogenic variants are associated with intellectual disability, dysmorphic features, hypotonia, and neurological impairments with similarities to Smith–Magenis syndrome

**DOI:** 10.1186/s13073-019-0630-1

**Published:** 2019-03-25

**Authors:** Francesco Vetrini, Shane McKee, Jill A. Rosenfeld, Mohnish Suri, Andrea M. Lewis, Kimberly Margaret Nugent, Elizabeth Roeder, Rebecca O. Littlejohn, Sue Holder, Wenmiao Zhu, Joseph T. Alaimo, Brett Graham, Jill M. Harris, James B. Gibson, Matthew Pastore, Kim L. McBride, Makanko Komara, Lihadh Al-Gazali, Aisha Al Shamsi, Elizabeth A. Fanning, Klaas J. Wierenga, Daryl A. Scott, Ziva Ben-Neriah, Vardiella Meiner, Hanoch Cassuto, Orly Elpeleg, J. Lloyd Holder Jr, Lindsay C. Burrage, Laurie H. Seaver, Lionel Van Maldergem, Sonal Mahida, Janet S. Soul, Margaret Marlatt, Ludmila Matyakhina, Julie Vogt, June-Anne Gold, Soo-Mi Park, Vinod Varghese, Anne K. Lampe, Ajith Kumar, Melissa Lees, Muriel Holder-Espinasse, Vivienne McConnell, Birgitta Bernhard, Ed Blair, Victoria Harrison, The DDD study, Donna M. Muzny, Richard A. Gibbs, Sarah H. Elsea, Jennifer E. Posey, Weimin Bi, Seema Lalani, Fan Xia, Yaping Yang, Christine M. Eng, James R. Lupski, Pengfei Liu

**Affiliations:** 1 Baylor Genetics, Houston, TX 77021 USA; 2 Northern Ireland Regional Genetics Service, Belfast City Hospital, Belfast, UK; 3 Department of Molecular and Human Genetics, Baylor College of Medicine, Houston, TX 77030 USA; 4 Nottingham Genetics Service, Nottingham City Hospital, Nottingham, UK; 5 Department of Pediatrics, Baylor College of Medicine, San Antonio, TX 78207 USA; 6 North West Thames Regional Genetics Service, 759 Northwick Park Hospital, London, UK; 7 Human Genome Sequencing Center, Baylor College of Medicine, Houston, TX 77030 USA; 8 Dell Children’s Medical Group, Austin, TX 78723 USA; 9 Division of Genetic and Genomic Medicine, Nationwide Children’s Hospital; and Department of Pediatrics, College of Medicine, Ohio State University, Columbus, OH 43205 USA; 10 Department of Pediatrics, College of Medicine & Health Sciences, United Arab University, Al Ain, UAE; 11 Department of Pediatrics, Tawam Hospital, Al-Ain, UAE; 12 Department of Pediatrics, Section of Genetics, University of Oklahoma Health Sciences Center, Oklahoma City, OK 73104 USA; 13 Department of Human Genetics and Metabolic Diseases, Hadassah-Hebrew University Medical Center, Jerusalem, Israel; 14 Department of Pediatrics, Texas Children’s Hospital, Houston, TX 77030 USA; 15 Department of Pediatrics, University of Hawaii, Honolulu, HI 96826 USA; 16 Centre de Génétique Humaine, Université de Franche-Comté, Besançon, France; 17 Department of Neurology, Boston Children’s Hospital, Boston, MA 0211 USA; 18 Gene DX, Gaithersburg, MD 20877 USA; 19 West Midlands Regional Clinical Genetics Service and Birmingham Health Partners, Women’s and Children’s Hospitals NHS Foundation Trust, Birmingham, UK; 20 East Anglia Regional Genetics Service, Addenbrooke’s Hospital, Cambridge, UK; 21 All-Wales Medical Genetics Service, University Hospital of Wales, Cardiff, UK; 22 South East of Scotland Clinical Genetic Service, Western General Hospital, Edinburgh, UK; 23 North East Thames Regional Genetics Service, Great Ormond Street Hospital, London, UK; 24 South East Thames Regional Genetics Service, Guy’s Hospital, London, UK; 25 Oxford Regional Genetics Service, Oxford University Hospitals, Oxford, UK; 26 Wessex Clinical Genetics Service, Princess Anne Hospital, Southampton, UK; 27 The DDD Study, Wellcome Trust Sanger Institute, Hinxton, Cambridge, UK; 28 The Hebrew University of Jerusalem, Jerusalem, Israel; 29 Monique and Jacques Roboh Department of Genetic Research, Hadassah-Hebrew University Medical Center, 91120 Jerusalem, Israel; 30 Present address: Department of Medical and Molecular Genetics, Indiana University School of Medicine, Indianapolis, IN 46202 USA; 31 Department of Molecular Physiology and Biophysics, Baylor College of Medicine, Houston, TX 77030 USA; 32 Present address: Mayo Clinic Florida, Department of Clinical Genomics, Jacksonville, FL 32224 USA

**Correction to: Genome Med (2019) 11:12**

**https://doi.org/10.1186/s13073-019-0623-0**

It was highlighted that the original article [1] contained a typographical error in the Results section. Subject 17 was incorrectly cited as Subject 1. This Correction article shows the revised statement. The original article has been updated.

**Correct statement:**

“Of note, subject 17 of our cohort presented with mild delayed motor milestones, generalized hypotonia, and, in particular, dysmorphic features including midface hypoplasia, tented upper lips, along with sleep issues, ASD, food-seeking behavior, and aggressive behavior; these clinical features are similar to those reported in SMS.”
