# Supplementary material for: De novo and inherited TCF20 pathogenic variants are associated with intellectual disability, dysmorphic features, hypotonia, and neurological impairments with similarities to Smith–Magenis syndrome
Source: Genome Med. 2019 Feb 28;11:12. doi: 10.1186/s13073-019-0623-0 (PMC6393995; doi:10.1186/s13073-019-0623-0)
Supplement: Supplementary file 1 — Clinical information. Clinical presentation of the subjects in this study. Table S1. Phenotypes for de-identified subjects from the DECIPHER database. Figure S1. Schematic representation of key conserved domains between TCF20 and RAI1. Figure S2. TCF20 alleles with premature termination codon variants escape from nonsense-mediated decay (NMD). (PDF 393 kb) [file 13073_2019_623_MOESM1_ESM.pdf]

De novo and inherited *TCF20* pathogenic variants are associated with intellectual disability, dysmorphic features, hypotonia, and neurological impairments with similarities to Smith–Magenis syndrome

Authors:

Francesco Vetrini[1,30] , Shane McKee[2], Jill A. Rosenfeld[3], Mohnish Suri[4] , Andrea M. Lewis[3], Kimberly Margaret Nugent[3,5], Elizabeth Roeder [3,5], Rebecca O. Littlejohn[3,5], Sue Holder[6], Wenmiao Zhu[1], Joseph T. Alaimo[3], Brett Graham[3,30], Jill M. Harris[8], James B. Gibson[8], Matthew Pastore[9], Kim L. McBride[9], Makanko Komara[10], Lihadh Al-Gazali[10], Aisha - Al Shamsi[11], Elizabeth A. Fanning[12], Klaas J. Wierenga[12,32], Daryl A. Scott[3,31], Ziva Ben-Neriah[13], Vardiella Meiner[13], Hanoch Cassuto[28], Orly Elpeleg[29], J. Lloyd Holder Jr[14], Lindsay C. Burrage[3], Laurie H. Seaver[15], Lionel Van Maldergem[16] , Sonal Mahida[17], Janet Soul[17], Margaret Marlatt[17], Ludmila Matyakhina[18], Julie Vogt[19], June-Anne Gold[20], Soo-Mi Park[20], Vinod Varghese[21], Anne K. Lampe[22], Ajith Kumar[23], Melissa Lees[23], Muriel Holder-Espinasse[24], Vivienne McConnell[2], Birgitta Bernhard[2], Ed Blair[25], Vicki Harrison[26], The DDD study[27], Donna M. Muzny[3,7], Richard A. Gibbs[3,7], Sarah H. Elsea[1,3], Jennifer E. Posey[3], Weimin Bi[1,3], Seema Lalani [1,3,14], Fan Xia[1,3], Yaping Yang[1,3], Christine M. Eng[1,3], James R. Lupski[1,3,7,14], Pengfei Liu[1,3]

1. Baylor Genetics, Houston, TX 77021, USA.
2. Northern Ireland Regional Genetics Service, Belfast City Hospital, Belfast, UK.
3. Department of Molecular and Human Genetics, Baylor College of Medicine, Houston, TX 77030, USA.
4. Nottingham Genetics Service, Nottingham City Hospital, Nottingham, UK.
5. Department of Pediatrics, Baylor College of Medicine, San Antonio, TX 78207, USA.
6. North West Thames Regional Genetics Service, Northwick Park Hospital, London, UK.
7. Human Genome Sequencing Center, Baylor College of Medicine, Houston, TX 77030, USA.
8. Dell Children's Medical Group, Austin, TX 78723, USA.
9. Division of Genetic and Genomic Medicine, Nationwide Children's Hospital; and Department of Pediatrics, College of Medicine, Ohio State University, Columbus OH 43205 USA.
10. Department of Pediatrics, College of Medicine & Health Sciences, United Arab University, Al Ain, UAE.
11. Department of Pediatrics, Tawam Hospital, Al-Ain, UAE.
12. Department of Pediatrics, Section of Genetics, University of Oklahoma Health Sciences Center, Oklahoma City, Oklahoma 73104, USA.
13. Department of Human Genetics and Metabolic Diseases, Hadassah-Hebrew University Medical Center, Jerusalem, Israel
14. Department of Pediatrics, Texas Children's Hospital, Houston, TX 77030, USA.
15. Department of Pediatrics, University of Hawai'i, Honolulu, HI 96826, USA.
16. Centre de génétique humaine, Université de Franche-Comté, Besançon, France.
17. Department of Neurology, Boston Children's Hospital, Boston, MA 0211, USA.

18. Gene DX, Gaithersburg, MD 20877, USA.
19. West Midlands Regional Clinical Genetics Service and Birmingham Health Partners; and Women's and Children's Hospitals NHS Foundation Trust, Birmingham, UK.
20. East Anglia Regional Genetics Service, Addenbrooke's Hospital, Cambridge, UK.
21. All-Wales Medical Genetics Service, University Hospital of Wales, Cardiff, UK.
22. South East of Scotland Clinical Genetic Service, Western General Hospital, Edinburgh, UK.
23. North East Thames Regional Genetics Service, Great Ormond Street Hospital, London, UK.
24. South East Thames Regional Genetics Service, Guy's Hospital, London, UK.
25. Oxford Regional Genetics Service, Oxford University Hospitals, Oxford, UK.
26. Wessex Clinical Genetics Service, Princess Anne Hospital, Southampton, UK.
27. The DDD Study, Wellcome Trust Sanger Institute, Hinxton, Cambridge, UK.
28. The Hebrew University of Jerusalem. Jerusalem, Israel.
29. Monique and Jacques Roboh Department of Genetic Research, Hadassah-Hebrew University Medical Center, Jerusalem 91120, Israel.
30. Department of Medical and Molecular Genetics, Indiana University School of Medicine, Indianapolis, IN 46202 USA (current affiliation).
31. Department of Molecular Physiology and Biophysics, Baylor College of Medicine, Houston, TX 77030, USA.
32. Present address: Mayo Clinic Florida, Department of Clinical Genomics, Jacksonville, FL 32224, USA

Clinical phenotype of subjects # 1-to-# 32

Subject #1 is a 3-year-old male with global developmental delay, speech delay, nocturnal epilepsy, mild hypotonia, dysmorphic features including coarse facies, multiple congenital abnormalities including duplicated left kidney, atrial septal defect, tethered spinal chord and dilated coronary artery. Dysmorphic features include mildly myopathic face, mild infraorbital hypoplasia, long eyelashes, bilateral eyebrow flare, thin lateral brows, wide nasal bridge, protruding ears, full lower lip, upper lip mildly tented overhanging lower lip and tendency to have open mouth. Family history indicate that the mother has intellectual disability and dysmorphic facial features. His height, weight, and FOC were within normal range. He had complete resection of a thoracic neuroblastoma. FISH for *MYCN* (2p23-24) gene amplification on mediastinal neuroblastoma

tissue showed evidence of an extra copy of the *MYCN* gene, consistent with aneuploidy of chromosome 2. No evidence of MYCN gene amplification was observed in any cells examined.

Subject #2 (DDD_ 269612) is a 14-year-old female with global developmental delay, hypotonia, intellectual disability, severe language disorder. She had a large build and mild dysmorphic features including frontal bossing, full cheeks, long fingers with a contracture of 5th finger, long toes, a wide carrying angle and joint hypermobility.

Subject #3 is a 20-year-old male presenting with congenital hypotonia and global developmental delay; he walked and started talking around 3 years of age. He has been diagnosed with high functioning ASD, anxiety, dyspraxia and ADHD requiring medication. Head circumference is around the 97th percentile with an IQ of 79-100. He has been noted to be clumsy with mild kyphosis. MRI and metabolic tests were normal.

Subject #4 is a 3.25-year-old male with ASD diagnosed at the age of 20 months, hypotonia, sensory processing disorder, visuo spatial perception difficulty, dyspraxia, history of sleep problems, mild dysmorphic features including triangular face, frontal bossing, with head circumference of 51 cm (around 99th percentile).

Subject #5 (DDD_277986) is a 8.5-year-old male with history of severe developmental delay, hypotonia, and feeding difficulties. Now at 8.5 -year-old he is presenting with moderate intellectual disability, possible ADHD with no formal diagnosis, and attends a special needs school. He had feeding problems previously which has improved. His current head circumference is 50.5cm (0.4th-2nd), weight is 20.1kg (2nd) and height is 106.4 ($<$0.4th). His symptomatic mother is reported to have had a history of feeding difficulties during early childhood and mild-moderate learning difficulties.

Subject #6 is a 10.2-year-old male with developmental delays, hypotonia and dysmorphic features. Detailed neurological examination revealed decreased strength in upper and lower extremities, distal and proximal hypotonia, also poor coordination. Furthermore, has been diagnosed with epileptic syndrome with complex partial seizures with intractable epilepsy. Developmental delays include expressive language, fine motor, and to lesser degree receptive language and gross motor skills. He also has poor growth velocity and present with remarkable short stature but with proportional weight. He has a history of pneumonia.

Subject #7 is a 10-year-old male with global developmental delay, autistic spectrum disorder, self-harming behaviors, significant anxiety, attention deficit hyperactivity disorder, poor muscular tone, refractory generalized non-convulsive epilepsy, mild dysmorphic features, tapered 5th fingers with minor cutaneous 2-3 to syndactyly. Most recent EEG in 2017 showed epileptic disorder with multifocal origin and EEG in 2013 showed atypical absence seizures. The family history indicates that the mother and maternal half-sister both have intellectual functioning disability the maternal half-sister also has attention deficit disorder. His height, weight, and FOC were within normal range.

Subject #8 (DDD_274421) is a 6.9-year-old male with global developmental delay, seizures treated with Valproate, ADHD, sleep disturbance, hyperacusis, and abnormal air whorl.

Subject #9 (DDD_267241) is 11.9-year-old with intellectual disability, specific language disorder, movement abnormality, microcephaly, feeding difficulties, ADHD, obsessive compulsive behavior, 5th toe clinodactyly, and inverted nipples. Dysmorphic features include brachycephaly and low-set ears.

Subject #10 (DDD_261665) is a 11.5-year-old female with global developmental delay, mild-to-severe intellectual disability, severe delay in receptive and expressive language, obsessive-

compulsive and food-seeking behavior, altered sleep pattern with frequent awakening, gross motor and fine skills delay, obesity, and joint hypermobility.

Subject #11 is a 3.9-year-old male diagnosed with congenital hypotonia with moderate distal spasticity, autistic spectrum disorder (ASD) diagnosed by Autism Diagnostic Observation Schedule (ADOS) and intellectual disability, failure to thrive and short stature. He is developmentally delayed including gross and fine motor and severe expressive/receptive language disorder. He has recurrent otitis media, frequent infections with monthly episodic fever, and recurrent pneumonia. In addition, he suffers from dysphagia. Facial dysmorphism include relative macrocephaly, deep-set eyes, mild coarsening, long philtrum, wide mouth with full lips, mildly tented upper lip, and hypertelorism. Lab studies identified platelet dysfunction. Previous MRI and other metabolic testing resulted normal; genetic testing, including chromosomal microarray, karyotype, and fragile X molecular analysis, were unrevealing.

Subject #12 (DDD_305239) is a 3-year-old female presenting with global developmental delay, ASD, language delay, sleep disturbance, and joint hypermobility. She had dysmorphic features including upslanted palpebral fissures, epicanthus, short nose, depressed nasal bridge, and short lingual frenulum.

Subject #13 is a 14-month old male with global developmental delay, laryngeal cleft, plagiocephaly, prominent mildly over folded ears with bilateral epicanthal folds and left-sided fifth finger clinodactyly.

Subject #14 (DDD_284745) is a 7.9-year-old female with mild developmental delay, hypotonia, motor coordination disorder, febrile seizure, mild intellectual disability, mild language delay, ptosis, drooling, dysmorphic features and 5th finger clinodactyly.

Subject #15 (DDD_274474) is 2.5-year-old female with global developmental delay, hypotonia, gait ataxia, dysmorphic features including brachycephaly, myopathic facies, depressed nasal bridge, anteverted nares, open mouth with downturned angle, high and narrow palate.

Subject #16 (DDD_285718) is a 6.5-year-old male with moderate developmental delay, hypotonia, mild intellectual disability, ASD, ADHD and strabismus.

Subject #17 is a 5.4-year-old girl with significant expressive/receptive speech delay with developmental delay and history of mild gross motor delay and hypotonia with mild facial dysmorphic features (midface hypoplasia). At the age of 5 year she had a vocabulary or no more than 10 poorly articulated words with a normal ABR by report. She could sign about 30 single words as well. She can follow single step commands and can hold a pen and scribble. Her mother also reports that she is "clumsy" and easily trips, her right foot "turns inward" when walking, and her right eye sometimes turns inward. She has some aggressive behaviors. She has trouble staying asleep despite long-term use of melatonin. She has also food-seeking behavior and lack of satiety. Her height, weight, and FOC are within normal range.

Subject #18 (DDD_286061) is a 9 -year-old female with intellectual disability, hypotonia, poor concentration, and hyperactivity. She sat at 18 months and walked at 2 ½-year-old. Other features include strabismus, joint laxity, dysmorphic features (high anterior hairline, large and tall forehead, low-set posteriorly rotated ears, narrow mouth and long philtrum).

Subject #19 (DDD_266071) is 10.2-year-old male with global delay, autism, seizures, clumsiness and hyperactivity (requiring Risperidone treatment) as well as faecal soiling as a young child. He presents with episodes of paroxysmal, possible dystonic movements of upper limbs, however the tone and reflexes are recorded as normal. In addition, he has tall stature, brachycephaly, high pitched voice, 5th finger clinodactyly, confirmed X-linked ichthyosis with a deletion of the STS locus on X – this is familial, and several normal family members are also affected.

Subject #20 is an 8-year-old male born to a consanguineous family with intellectual disability, motor delay and insomnia. He walked at 1.5-year-old and started talking at 2-year-old. Examination at 3.5 years showed no dysmorphic features and no abnormal neurological signs. His weight was on the 90th centile till the age of 3 years when it dropped to the 50th and by the age of 8 years it dropped to <25th centile. His height and head circumference have been on the 75th centile throughout. At the age of 8 years he was reported to have slow movements, difficulty in swallowing, excessive salivation and started having blurred speech. Neurological examination revealed slightly increased tone at the elbows, normal deep tendon reflexes and impaired coordination.

Subject #21 (DDD_304759) is 33.3-year-old with moderate intellectual disability, tall stature and high-pitched voice.

Subject #22 (DDD_299802) is a 9.9-year-old female with severe intellectual disability, hypotonia, jerky movements, macrocephaly and overgrowth. At 12 years 3 months her height was 174.5 cm (99th percentile) and weight 81.5kg. Her dysmorphic features include long face with tall forehead, full cheeks, prominent lower lip and chin, sunken eyes.

Subject #23 (DDD_304749) is a 4-year-old male with global developmental delay, ASD, inverted nipples, dysmorphic features including broad forehead, short nose, and depressed nasal bridge.

Subject #24 (DDD_300882) is 7.8-year-old with global developmental delay, ASD, seizures, and low-set ears.

Subject #25 is 5.3-year-old female diagnosed with hypotonia, global developmental delay, severe speech disorder and minor facial dysmorphic features. She has quite marked hypotonia and developed relatively slow. She started rolling over at about 10 months of age with cruising and

crawling at 15 months. She spoke her first words at about 14 months of age and her vocabulary was only 4-5 words at the age of 18 months. Neurological examination did not reveal any other sign or symptoms. Her height, weight, and FOC were within normal range. She has sleep problems and needs melatonin and clonidine to sleep. She has hypohidrosis.

Subject #26 (DDD_261626) is a 3.1-year-old female with global developmental delay, hypotonia and ASD. MRI revealed delayed CNS myelination and lack of cerebral white matter. Dysmorphic features include long eyelashes, down slanting palpebral fissures, epicanthic folds, everted lower lip, open mouth, tapered finger.

Subjects #27 and #28 (DDD_294521) are two identical male twins with moderate intellectual disability, speech delay, hypotonia, motor coordination difficulties, plagiocephaly, bilateral ptosis, horizontal crus of helix, malar flattening, narrow mouth, short, tapering fingers with incurved 5th fingers, mild bilateral hallux valgus. In addition, they both present with inverted nipples, truncal obesity, and subject #28 has high arched palate. The family history indicates that the father presents with similar phenotype.

Subject #29 was initially evaluated at 4-year-old. She presented with congenital generalized hypotonia, repetitive behavior, and severe mixed expressive/receptive language disorder. She was adopted. Her prenatal history indicates that she was diagnosed with “brain damage syndrome” but no other information was provided. At the time of her adoption, mother noted scars on her legs that appeared to be surgical in nature. In addition, she showed stereotypy as a 6-year-old: hand flapping, walking in circles, hitting her head when frustrated. She was diagnosed with autism spectrum disorder, intellectual disability and inattentive type ADHD. Her height, weight, and FOC were within normal range.

Subject #30 is a 14-year-old male with global psychomotor delay, autism spectrum disorder, stereotypical behavior, severe language delay, macrocephaly, congenital hypotonia, and scoliosis.

He walked when he was older than 2 years of age and started talking in single words when he was 4 years old. He has had disrupted sleep patterns since infancy. Cranial imaging studies included a head CT that showed stable prominence of extra axial fluid spaces and MRI showed a slight asymmetry of the cerebral hemisphere. The picture in Fig. 2L was taken at 22 years old.

Subject #31 is a 5-year-old male with global developmental delay, intellectual disability, seizure and balance disorder. Macrocephaly 37 cm (+2SD) was noted at birth with. At 4 years, the height at +2SD. At 6 years, overgrowth was noted with a height of 120 cm (+2.5DS), a weight of 23.7 Kg (+2SD), and an OFC of 52.5cm (+1SD).

Subject #32 is a globally delayed 13-month- old female with severe receptive/expressive language delay, hypotonia, mild spasticity in ankle dorsiflexors, and mild proximal weakness in shoulders/hips. Her facial features include macrocephaly, midface hypoplasia, long eyelashes, thick lips and an occipital groove. At the last medical examination, she had marked language delay and emerging autistic traits. She displays food-seeking behavior and has sleep disturbance. Brain MRI showed mildly enlarged extra-axial spaces, lateral ventricles and large cavum septum pellucidum and vergae. Additional clinical findings include a renal cyst, GERD, dysphagia and delayed visual maturation.

Table S1. Phenotypes for de-identified subjects from the DECIPHER database

DECIPHER ID	Genotype	Phenotype
251708	1.22Mb *TCF20* deletion	Delayed speech and language development, Intellectual disability, Seizures
339549	*De novo* 1.22Mb *TCF20* deletion	Atrial septal defect, Cerebral cortical atrophy, Esotropia, Feeding difficulties, Generalized hypotonia, Global developmental delay, Mild microcephaly, Mild short stature, Patent ductus arteriosus
251299	*De novo* 3.80Mb *TCF20* deletion	Hypothyroidism, Intellectual disability, Slender build, Slender finger, Sparse and thin eyebrow, Ventricular septal defect
248554	*De novo* 1.67Mb *TCF20* deletion	Asymmetry of the ears, Clinodactyly of the 5th finger, Delayed speech and language development, Epicanthus,

		Facial asymmetry, Intellectual disability, Posteriorly rotated ears, Telecanthus, Up slanted palpebral fissure
274092	*De novo* 1.31Mb *TCF20* deletion	Delayed speech and language development, Feeding difficulties in infancy
259449	442kb *TCF20* deletion	Delayed speech and language development, Down slanted palpebral fissures, Macrocephaly, Plagiocephaly, Tall stature
257430	*De novo* 3.00Mb *TCF20* deletion	Intellectual disability, Macrocephaly
262531	*De novo* 1.78Mb *TCF20* deletion	Intellectual disability, Nystagmus, Sensorineural hearing impairment
255045	277kb *TCF20* deletion	Phenotype unavailable
256216	*De novo* 2.34 Mb *TCF20* duplication	Intellectual disability, Non-midline cleft lip, Short stature
258948	1.61 Mb *TCF20* duplication with other CNVs	Abnormality of the small intestine, Hypertelorism, Low-set ears, Micrognathia, Ventricular septal defect
1459	*De novo* 2.77 Mb *TCF20* duplication	Phenotype unavailable
269435	995kb *TCF20* duplication	Clinodactyly of the 5th finger, Delayed speech and language development, Epicanthus, Hypertelorism, Intellectual disability, Postaxial hand polydactyly, Seizures, Strabismus
255992	453kb *TCF20* duplication with other CNVs	Phenotype unavailable

Fig S1.

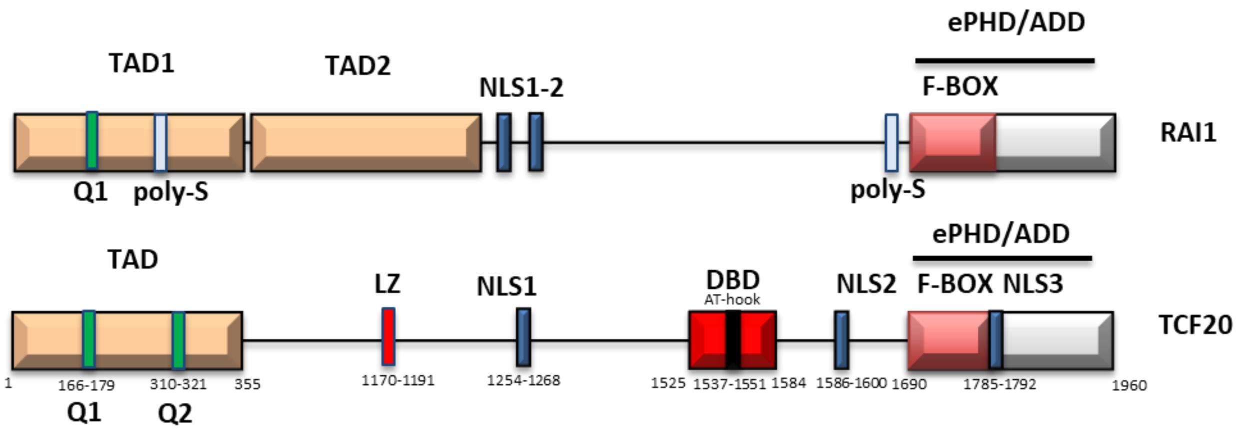

Fig. S1. Schematic representation of key conserved domains between TCF20 and RAI1. Functional domains of TCF20 (shared with RAI1) include TAD, Glutamine-rich stretches (Q), NLS1 and 2, F-BOX and ePHD/ADD. Additional unique domains present in TCF20 are LZ, NLS3 and DBD domains with the AT-hook region. The relative amino acid position in TCF20 is shown below the structure.

Fig S2.

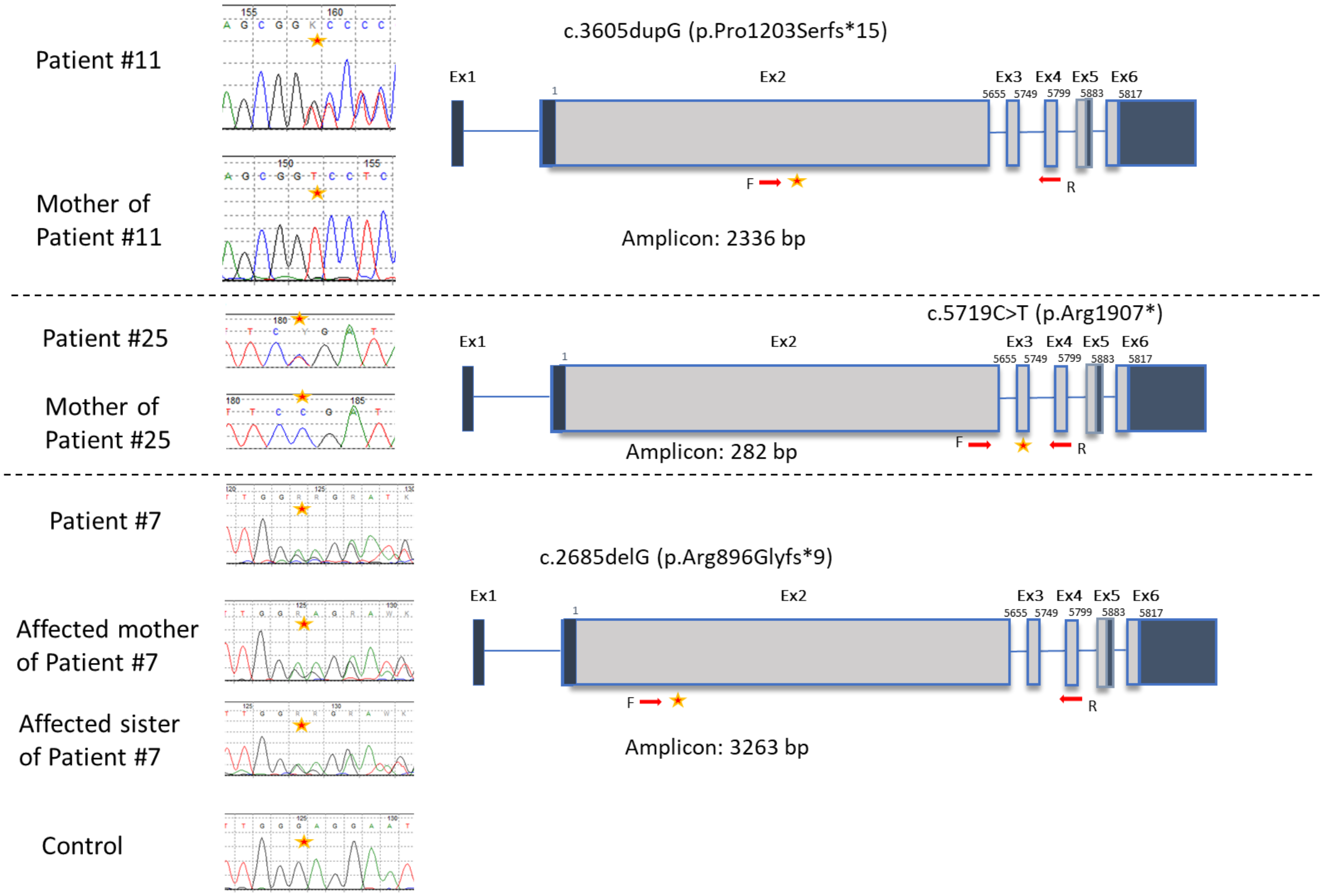

Fig. S2. *TCF20* alleles with premature termination codon variants escape from nonsense mediated decay (NMD). The mRNA of mutant *TCF20* are subject to reverse transcription and PCR. The PCR primers are designed so that the amplicons all span the intron 2 and 3 of *TCF20* (~30 kb and ~10 kb, respectively). This facilitates to minimize interference of genomic DNA from PCR. The locations of the primers are indicated on the genomic sequence of *TCF20*. The primer sequence are as follows. Patient #11 forward 5'-CAGTGGGGACTTACCATGACCC-3', reverse 5'-CGAGAAGTTCTCCTCATGTAGCAAAC-3'. Patient #25 forward 5'-AGCTGGAGTTACAAATCCCTGA-3', reverse 5'-TAGGGCACCTCACCGAGAA-3'. Patient #7 forward 5'-TTGAAATAGAGCCTCAGTCATCAGCAC-3', reverse 5'-GTGCTTAGGGCACCTCACCGAGAA-3'. The expected amplicon sizes from cDNA amplification are indicated in the figure. The sizes of the amplified segments are verified to ensure that no genomic DNA is present. Sanger sequencing chromatograms are illustrated. Individuals with a premature termination codon variant, including patient #11, #25, #7, and the mother and sister of patient #7, are expected to be subject to NMD by the '50 bp penultimate exon' rule. However, they all show a pattern that the mutant allele is of similar intensity to the wildtype allele, suggesting that the mutant mRNA escaped from NMD. Sequencing results from individuals without a *TCF20* variant, including the mother of patient #11, mother of patient #25, and an unrelated individual, are included as negative controls.
